# Bayesian Model Selection to Investigate Meaningful Spatial Scales

**DOI:** 10.1002/ece3.73271

**Published:** 2026-04-08

**Authors:** Andrew Hoegh, Kathryn M. Irvine, Katharine M. Banner, Luz de Wit, Brian Reichert

**Affiliations:** ^1^ Department of Mathematical Sciences, Montana State University Bozeman Montana USA; ^2^ Centre for Planetary Health and Food Security, Griffith University Nathan Australia; ^3^ Northern Rocky Mountain Science Center, U.S. Geological Survey Bozeman Montana USA; ^4^ Bat Conservation International Austin Texas USA; ^5^ Fort Collins Science Center, U.S. Geological Survey Bozeman Montana USA

**Keywords:** Bayes factors, ecological statistics, reversible jump MCMC, spatial ecology

## Abstract

Ecologists and other statistical practitioners with access to high‐resolution spatial data often lack guidance on best approaches for discerning meaningful spatial scales for environmental covariates which is necessary when spatial factors influence environmental processes. Recently developed methods have attempted to automate investigating spatial scales for covariates by evaluating models for which potential explanatory variables are derived from circular extents of increasing size centered at survey locations. However, these methods make a strong assumption on the inclusion of the covariate and do not help discern whether a covariate should be included in the model. We present an approach that utilizes researcher guidance to create informative priors on the model space that, along with parallelizable Reversible Jump Markov chain Monte Carlo techniques, enables efficient estimation of posterior model probabilities to assist with the choice of meaningful spatial scales for environmental covariates.

## Graphical abstract

Ecologists and other statistical practitioners with access to high‐resolution spatial data lack guidance on best approaches for discerning meaningful spatial scales for environmental covariates, which is necessary when spatial factors influence environmental processes. We present an approach that utilizes researcher guidance to create informative priors on the model space that enables efficient estimation of posterior model probabilities to assist with the choice of meaningful spatial scales for environmental covariates.

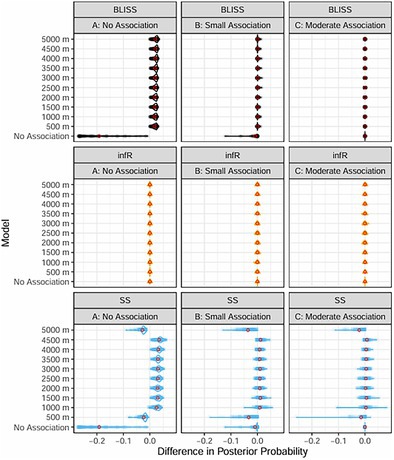

## 1 Introduction

Model selection is fundamental to making inferences on spatial ecological processes that inform environmental management decisions.^1^ In these settings, when investigating environmental management decisions, variables may influence an ecological observation at an unknown spatial scale. Thus, research in ecology often relies on considering multiple models corresponding to the spatial scales, which are formulated using existing knowledge of the system and compared based on how the data support each model. Researchers are faced with having to construct models by selecting among ecologically relevant but likely highly correlated environmental covariates. A model selection approach that can guide this process by informing which environmental covariates are related to the quantity of interest could help inform environmental management decisions.

In landscape ecology, understanding a species' habitat requirements for continued persistence is needed to inform how large an area from known critical locations should be considered when establishing protection areas or habitat mitigation strategies (Fahrig 2001). An approach for examining spatial scales of association for covariates is the Frishkoff et al. (2019) scale selection (SS) method, which was originally formulated in an occupancy model setting, but has since been adapted and implemented in other works (Monroe et al. 2022). With SS a matrix of spatial covariates, containing values calculated at increasing circular spatial extents (e.g., every 50 m), would be evaluated using an indicator variable that controls which spatial extent is currently active. Generally, the prior on this indicator variable is a discrete uniform distribution designed to index columns of the matrix. Another approach is Bayesian latent indicator scale selection (BLISS) (Stuber et al. 2015), which also uses an indicator variable on prespecified spatial extents. The extents can be specified by the researcher but are often more coarse than the automated approach with SS. As generally configured, BLISS requires at least one scale to be included in the model. These approaches show promise for exploring spatial extents associated with covariates that have a known relationship with the ecological response of interest; however, as commonly used, when the relationship between a covariate and response is unknown or debated by experts, neither approach has the ability to consider that a covariate has no relationship with the response.

We present a fully Bayesian model‐based model selection procedure that uses researcher knowledge to guide the choice of informed priors on the model space and investigate spatial scales of association between a covariate and response of interest. This alternative approach is tailored to not only infer the spatial extent but also explore whether the covariate has a relationship with the response of interest. This approach, which we implement using an efficient Reversible Jump Markov Chain Monte Carlo (RJMCMC) algorithm (Barker and Link 2013), we will refer to as “infR” based on our approach using informed priors and the algorithm. This approach requires explicit choice and justification of the model sets and associated priors (Grace and Irvine 2020); consequently, a model and prior weight that does not include the spatial covariate can be incorporated into the framework enabling a weaker assumption on the structure while also controlling for multiple comparison issues via Bayesian prior model probabilities (Neath et al. 2018), when screening a large number of spatial covariates.

There are many competing philosophies on model selection for understanding spatial scales in ecology. Some methods estimate a continuous response over spatial extents for a specified covariate (Miguet et al. 2017; Yeiser et al. 2021) whereas others, such as our approach and SS and BLISS, use model‐based model selection approaches, as defined in Hooten and Hobbs (2015), where each extent corresponds to a discrete model. Still, other traditional statistical paradigms for model selection, such as horseshoe priors (Carvalho et al. 2009) and other local–global shrinkage priors, can be applied to this type of setting. The continuous extent approaches can be useful if a covariate has a known relationship with an ecological response at some spatial extent. In this work, we assume that researchers have an absence of knowledge or a lack of consensus regarding whether a spatially explicit variable should be considered at all, consistent with a purely exploratory investigation. Our approach, which allows for prior specification on the model space, could be used as a screening tool and part of the EDA phase within an iterative, model‐building procedure such as that highlighted in Ver Hoef and Boveng (2015). Thoughtful prior structure, including prior mass on models without spatial covariates, can control for potential errors when considering large numbers of predictors.

## 2 Background on Bayesian Model Selection

With model‐based model selection approaches, researchers compare different models with the goal of investigating posterior model probabilities, which are expressed as $\mathrm{Pr}\left[M_{k}|Y\right]$ where $M_{k}$ is the $k^{\mathrm{th}}$ model and Y represents the data. Posterior model weights also form the basis of model averaging (Hoeting et al. 1999); however, we exclude these techniques as they are more suited for predictive settings rather than exploratory settings which are the focus of this work.

Posterior model probabilities can be calculated as follows:

(1) $$\mathrm{Pr}\left[M_{k}|Y\right]=\frac{\mathrm{Pr}\left[M_{k}\right]\times p\left[Y|M_{k}\right]}{p\left(Y\right)}$$

where the term $p\left[Y|M_{k}\right]$ is the marginal likelihood and requires integrating out the model parameters such that

(2) $$p\left[Y|M_{k}\right]=\int p\left(Y|\theta ,M_{k}\right)p\left(\theta |M_{k}\right)\mathrm{d\theta}.$$

Computing posterior model probabilities requires two layers of prior distributions. The first layer of prior distributions are placed on model parameters in Equation (2), which are denoted as $p\left[\theta |M_{k}\right].$ The second layer of priors, denoted as $\mathrm{Pr}\left[M_{k}\right],$ are placed on the models and generally are expressed as a set of probabilities that sum to one.

Bayesian algorithms are used to approximate the integration in Equation (2) and Equation (1) to obtain posterior model probabilities. There are two fundamental types of these algorithms: transdimension approaches, namely reversible jump (Green 1995), and indicator type approaches (Kuo and Mallick 1998; George and McCulloch 1993). With transdimensional approaches, MCMC transitions are proposed across model spaces of potentially different dimensions, which require a bijection function to account for the different model dimensions. In applications investigating the spatial scale for species habitat requirements, the dimension is governed by the number of coefficients for environmental covariates. With indicator approaches, the dimension of the model spaces is constant, but indicator variables are used to specify whether a variable is active in the current iteration of the algorithm. When variables are not active, samples are still drawn from pseudo‐priors for those coefficients. Regardless of the approach, model selection algorithms can be difficult to specify and fit. For a comprehensive overview of the numerous Bayesian model selection algorithms, readers are referred to O'Hara and Sillanpää (2009) and Piironen and Vehtari (2017).

The extra challenge in this setting, when considering spatial covariates on increasing circular extents, is that the covariates will be highly correlated. This poses challenges for many of the model‐based model selection algorithms, although there are some tailored for this purpose (Kwon et al. 2011; Ghosh and Ghattas 2015; Hoegh et al. 2018). Rather, we implement a parallelizable RJMCMC algorithm presented by Barker and Link (2013). The benefit of this implementation of a reversible jump approach is that each model can be fit separately, in parallel, and then combined; thus avoiding posterior convergence issues related to highly correlated predictors.

## 3 Model Framework

Our goal is to present a framework for investigating whether spatial covariates have a relationship with a response of interest when the relationship is unknown a priori or there is disagreement among the experts and, if so, exploring the spatial extent of a covariate that explains the response of interest. As part of that process, eliciting expert opinion through informative priors is highly valued to inform plausible models and should require justification of model sets (Grace and Irvine 2020) and associated priors corresponding to extents representing the hypothesized spatial scales of association of a species‐habitat association.

### 3.1 Prior Elicitation

Although specifying prior distributions is a fundamental component of Bayesian analysis, prior distributions are often selected in a default manner that lacks scientific justification or are otherwise disconnected from researchers' knowledge but rather are the result of default values in software tools. There are many challenges with prior elicitation which attempts to map researcher domain knowledge into probability distributions (Mikkola et al. 2023). However, given the discrete nature of prior model probabilities, this is a situation that may be well suited for interactive prior elicitation.

**FIGURE 1:**
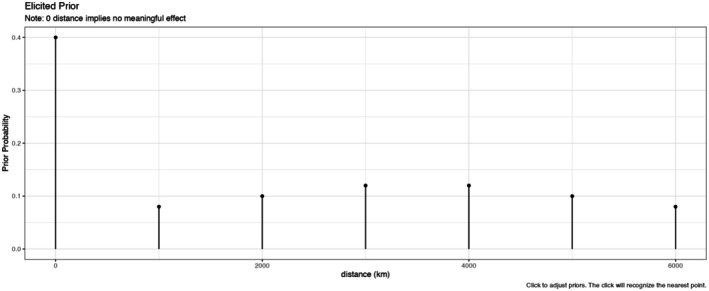
Example of an elicited prior on extents. There is a prior probability of 0.4 that the variable is not meaningful. The remaining 0.6 of the prior mass is allocated across the six spatial extents with the most likely distances being in the middle of the selected range.

We have created an R package that contains a Shiny app (Chang et al. 2024) to accompany this work that can be installed directly from GitHub (BozemanEnvrStat/infRJ); the Shiny app helps elicit prior model probabilities associated with extents for the covariate. These model probabilities feed into our modeling framework to investigate extents for spatial covariates. Figure 1 contains an example of a prior distribution on models associated with the extent that can be generated from the Shiny app. In this example, there is a point mass at zero of 0.4, which corresponds to the prior probability that the covariate is not meaningful. The rest of the prior mass is placed at 1000 to 6000 m spatial extents. Given that the researchers can select the spatial extents to consider, it is reasonable to assume that the extents toward the middle are more likely, a priori; otherwise, the researcher could include extents associated with larger distances to include the largest possibly hypothesized scale with even larger extents to be safe. Accordingly, this prior includes greater prior mass for the extents in the middle of the selected range. This prior corresponds to seven discrete potential models to explore, one of which does not include the covariate.

There are alternate ways to use the app to choose a prior distribution. The Shiny app can automatically generate evenly spaced extents or users can also directly input extents along with prior probabilities. With either option, the probabilities can be adjusted by interactively clicking on the plot to increase or decrease prior probability at a point, which will normalize beliefs to correspond to a probability distribution. There is no restriction that prior mass is uniformly placed on extents or that identified extents are evenly spaced as the app will normalize them to sum to one. With the prior in Figure 1, we are intentionally not considering models defined by covariates derived from models at very close extents. Rather, 1000 m increments are considered to be a meaningful distance for evaluating the various spatial associations between a covariate and biological response.

### 3.2 Computation

Given a model set and associated priors, as depicted in Figure 1, the goal is to estimate the posterior model probabilities from Equation (1). We implement Barker and Link (2013)'s reversible jump algorithm. The idea with this Gibbs sampling‐based RJMCMC algorithm is that each model can be run in parallel and then those individual posterior samples are combined to estimate posterior model probabilities. This may be especially advantageous when there is a high degree of correlation between variables in the model sets—caused here by the similarity of geospatially derived characteristics from increasing circular extents.

In addition to running standard MCMC to estimate parameters in each individual model, Barker and Link (2013)'s RJMCMC algorithm also requires simulating, in the author's words, a complete palette of parameters. In this case, the palette of parameters requires that posterior samples associated with all spatial extents are simulated for each model. In combining MCMC samples from across the set of models, the algorithm uses a Rao‐Blackwell approach to estimate posterior model probabilities. However, convergence of the chains for individual models is not sufficient to guarantee convergence of the posterior model probabilities. Rather, this convergence is a function of the number of models in the set, the number of iterations, and the pseudo‐priors that are used to generate samples for the palette of parameters that correspond to environmental covariates that are not active in that particular model. In simple univariate screening settings, convergence of the individual models is generally sufficient; however, for more complicated and higher dimensional model sets, extra attention should be given to the posterior probabilities. Thus, the convergence should be monitored such that an acceptable level of Monte Carlo error in the model posterior probabilities is present. Most MCMC algorithms run multiple chains with default settings, so the user can verify convergence for the individual models and examine the Monte Carlo error by computing posterior probabilities across the different sets of chains. For simulations, we used between 3000 and 5000 iterations for the underlying models. Nevertheless, this approach avoids the necessary complications of directly transversing highly correlated model spaces via traditional reversible jump or using indicator approaches.

## 4 Simulation Study: Model Comparison

Although our framework (infR), with an informed prior over the model space, has some fundamental differences from the state‐of‐the‐art methods SS and BLISS, we can make some comparisons in our simulation study. We use a scenario where the variance term is assumed known in a linear model so that the Bayes factors and true model probabilities can be analytically calculated, resulting in a gold standard for comparison. Additional theoretical details are available in the Appendix 1.

For the simulation, the strength of the “true” signal varies from moderate to weak to no signal. To mimic a realistic environmental covariate, we use coordinates from 100 bridges that represent potential bat roosts (Oram et al. 2025). For each of the 100 locations, we extract the proportion of land classified as developed (Homer et al. 2012) around the point location with 10 circular extents of increasing radii from 500 m to 5000 m in 500 m increments. The correlation between these environmental covariates ranges from about 0.85, for 500 m and 5000 m, to close to 1 for many extents separated by only 500 m. These same covariate values are used across 100 replications where a “true model” is established as $y=\beta_{0}+\beta_{5}x_{2500}+\epsilon$, where $\epsilon ∼N\left(0,1\right)$, $\beta_{0}$ is 1 for all simulations and the value of $\beta_{5}$ varies across the three simulation scenarios from 0, 1, and 1.8 for “no association,” “weak association,” and “moderate association,” respectively. In this simulation, y is a generic response measured at the center of the circular extents. By default, there is no direct relationship between the remaining covariate extents, say $x_{1000}$, and the response of interest.

We assume a uniform prior across the model space. This manifests differently across the models' as BLISS (Stuber et al. 2015) and SS (Frishkoff et al. 2019) do not explicitly have a way to put prior mass on the “no‐association” model, because these models are more configured to identify the best spatial extent assuming a covariate has a known or previously documented relationship. The prior distribution for all of the β values is a standard normal prior, which is weakly informative in this setting. For this simulation, all three models have relatively similar run time with a single replicate of the simulation study taking less than 1 min.

**FIGURE 2:**
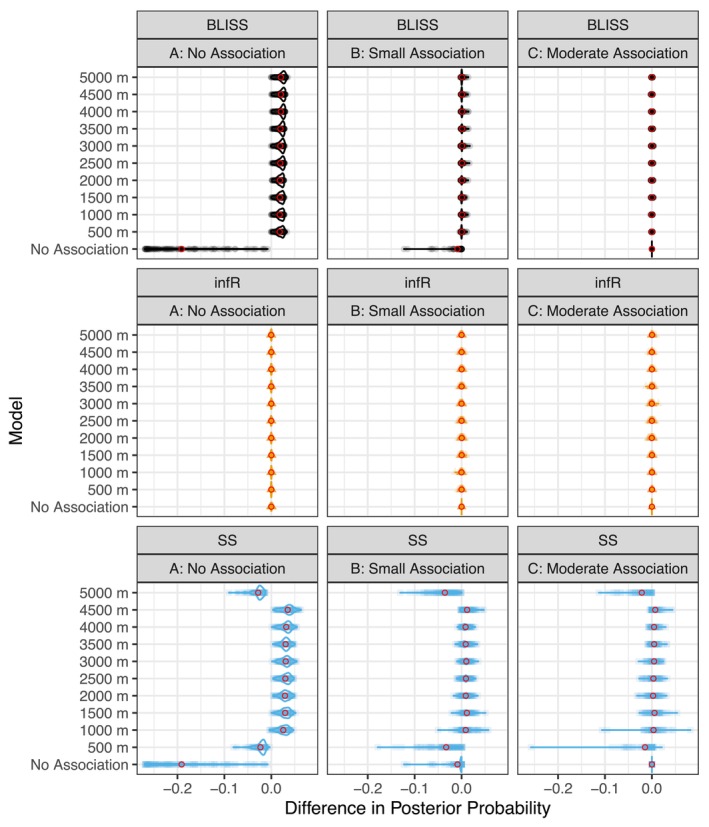
Differences in estimated and true posterior model probabilities are displayed with 100 replications for each setting where red circles represent the average difference. Across all settings, our modeling approach (infR) has accurate and precise estimates. Because BLISS and SS do not have a mechanism for evaluating a model with no explanatory variable, both struggle with no association and a small association. BLISS does is more accurate with a moderate association, but SS still performs relatively poorly.

As seen in Figure 2 and Table 1, infR has the highest performance compared to both SS and BLISS in the no association and small association situations. Due to the inability of these frameworks to consider a model without a known relationship with the covariate, the posterior model probabilities for the other models are biased and result in too large estimates. Furthermore, SS suffers from some edge effects and struggles with estimating posterior model probabilities on the smallest and largest extents considered. In the moderate association setting, where the posterior model probability of the no association model is small, BLISS and our method perform similarly (Table 2).

**TABLE 1:** Mean absolute deviation and root mean square error between true model posterior probability and model estimates for the simulation. The infR model gives more accurate estimates of the model posterior probabilities.

Model	MAD	rMSE
BLISS	0.013	0.039
SS	0.024	0.045
infR	0.001	0.002

**TABLE 2:** Mean absolute deviation and root mean square error between true model posterior probability and model estimates for the three associations in the simulation. The infR model gives more accurate estimates of the model posterior probabilities for setting A: No Relationship. For the small and moderate relationships, infR and BLISS are similar and both are more accurate than SS.

Association	mad_BLISS	mad_SS	mad_infR	rmse_BLISS	rmse_SS	rmse_infR
A: No	0.035	0.044	0.001	0.065	0.069	0.001
B: Small	0.002	0.015	0.001	0.007	0.023	0.002
C: Moderate	0.001	0.010	0.001	0.001	0.019	0.002

## 5 Synthetic Data Case Study: Screening Tool

We also present a case study to demonstrate how this framework can be used as a univariate screening tool. Similar to the simulation, we select coordinates for 100 sites that correspond to bridges which could potentially serve as roosts for bats. At each location, we calculate the proportion of land categorized in the eight main National Land Cover Database (NLCD) classes (Water, Developed, Barren, Forest, Shrub, Herbaceous, Planted/Cultivated, Wetlands) across 10 extents from one kilometer to 10 km.

We simulate data from a model where only one variable, Forest at 5 km, is explicitly included in the model. Due to the data structure, this still results in very high correlation with percent forested coverage at other extents where the correlation ranges from 0.92 to 0.99. Additionally, this results in correlation up to about with the proportions of the other cover classes at all spatial extents being considered. Specifically, we use a similar framework to previous simulations where σ=1 and the β value associated with percent forest coverage at 5 km is 8. For reference, this results in an $R^{2}$ value of approximately 0.65.

For the case study, we screen all eight variables, each at 10 extents. Our goal is to inform which variables are meaningful and their corresponding zone of influence. Note, that the univariate screening approach reduces the dimension of the model space considerably. Rather than considering all 11^8 (~200 million) models jointly—which would be impractical in either a stochastic search or parallelized reversible jump setting—we use the same prior on the model space to screen all of the variables. In particular, this prior places half of the prior mass is placed on the null model corresponding to not including that particular variable and the remaining half of the prior mass is allocated in a stair step framework with values of $\frac{1}{60}$, $\frac{2}{60}$, $\frac{3}{60}$, $\frac{4}{60}$, $\frac{5}{60}$, $\frac{5}{60}$, $\frac{4}{60}$, $\frac{3}{60}$, $\frac{2}{60}$, and $\frac{1}{60}$ corresponding to the extents from 1 to 10 km. This nonuniform prior on model space corresponds to user belief that distances in the middle of the selected range are most likely a priori.

**FIGURE 3:**
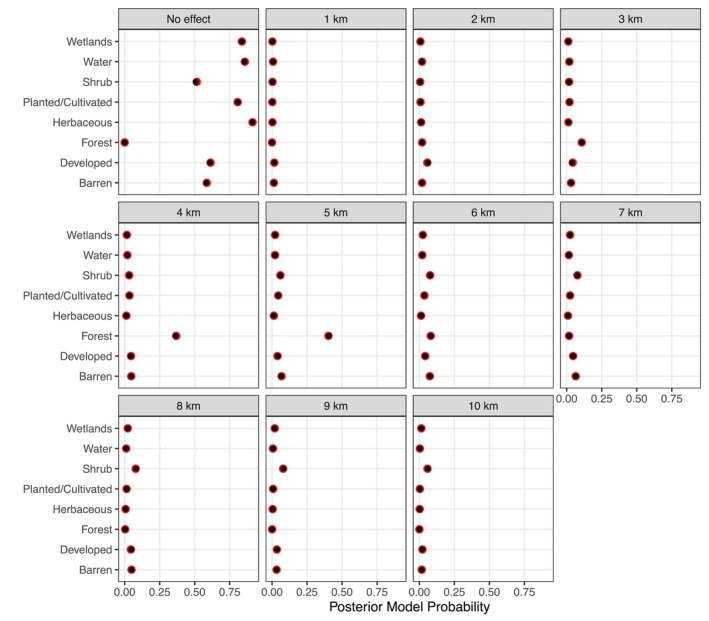
Posterior model probabilities for each variable across the 11 model possibilities in the case study. The red circles are the true posterior probabilities and the black circle are the model estimates. As we would expect, for all models except the Forest land use, there is strong support for the model with no effect. With the forest percent coverage, the posterior mass is spread across models from 3 to 6 km, with a majority of the mass on 4 km or 5 km.

Assuming that the variance is known allows exact calculations for the posterior model probabilities associated with each model. With the specified priors, the exact posterior model probabilities can be seen in Figure 3. For the seven variables that are not in the model, the posterior probability of association is always at least 0.5. For some of these variables that are highly correlated with forest cover, the values are close to 0.5. For forest cover, the variable used to simulate the synthetic data, the posterior probability of no association is virtually zero, whereas the posterior probability is the highest, with a value of 0.40, on the association at the 5 km extent—the true value from the simulated model.

Figure 3 also contains posterior probabilities for all eight variables included in this synthetic data case study. This information would likely encourage researchers to focus on models containing forest cover. The highest posterior value would be at the 5 km spatial extent, but there is also strong support, with a posterior probability of 0.37, for a 4 km spatial extent. In this framework, researchers could further investigate the variables for shrub, developed, and barren, but the posterior model probability for no association is roughly an order of magnitude greater than the posterior support for a model at any specific spatial extent. If multiple spatial variables were identified in the screening procedure, modeling could follow a standard, post exploratory data analysis workflow for considering those variables and interactions.

As demonstrated with the simulation study, our model framework returns precise estimates of the posterior model probabilities. When model variance terms are unknown, the exact posterior model probabilities cannot be empirically calculated and a model‐based approach, such as what we have presented here, would be necessary to estimate posterior model probabilities. The model framework, and the associated reversible jump framework, could easily be applied to more complicated model settings including generalized linear models or occupancy models that are common in ecological studies.

## 6 Discussion

Bayesian methods provide a useful approach to advancing the understanding of scale dependency in ecology (Stuber and Gruber 2020). Here, we provide a tool that leverages Bayesian model selection to enhance statistical methods that inform environmental management and improve upon existing methods. This tool allows researchers to explore the spatial scales of association for environmental covariates, which is key in ecological research where spatial dynamics influence environmental processes. This approach supports environmental policy and management decisions and strategies based on the interpretation of ecological dynamics occurring at appropriate spatial scales. Compared to existing methods, which are better formulated to identify the scale at which a covariate is meaningful, assuming a relationship between the covariate and response is known, our approach aids in exploratory investigation when it is unclear if the covariate has any relationship with the response and performs at least as well as existing methods in the presence of a strong signal. Moreover, we provide a fully integrated R workflow that enables users to extract geospatial information, elicit informative prior information, and estimate posterior prior probabilities using efficient parallelizable RJMCMC.

Our informed model selection procedure is best used for exploration or toward increasing explanatory understanding, as opposed to prediction. We envision the most common approach would be a lower dimensional screening tool, as in our case study, in the exploratory phases of an iterative model building procedure like that highlighted in Ver Hoef and Boveng (2015). Thoughtful choice of priors on the model space, particularly mass on models without spatial covariates, can help avoid multiple comparison issues when considering a high number of model sets. The examples presented here are focused on simple model specifications—linear models where the variance is assumed known. Estimating variance terms in the linear model frameworks will have minimal impacts on model convergence; however, eliciting priors over more complicated model settings, even just lower dimension frameworks with potential priors, can be very difficult. In those cases, iterative model building procedures can ease some of those challenges, and our framework can play a vital role in that process.

Although our goal is primarily to aid in the exploration of relationships between the spatial covariates and the response of interest during the exploratory phase of an analysis, if it is known that a spatial covariate is meaningful—either using this method or otherwise—there are many valid alternative approaches. Both SS and BLISS were developed to be used in this setting. Additionally, another option is to treat the spatial extent of the association in a continuous manner rather than the discrete nature used by our approach or SS and BLISS; Miguet et al. (2017) provides a distance‐weighted method, rather than from a threshold‐based approach. This is fundamentally different from estimating the maximum effective scale for a spatial covariate, but instead uses a weighted approach over the spatial extent whereas threshold methods, including ours, weight everything in the spatial extent equally. For predictive settings, a distance‐weighted approach might be preferred but may have a more difficult scientific interpretation. Nevertheless, using our framework as a screening tool to explore spatial covariates does not prohibit more sophisticated model approaches like distance‐dependent modeling approaches detailed in Yeiser et al. (2021); rather, they could be used in an iterative model building framework where our approach is used to screen spatial covariates and explore important ones to be used in other algorithms to construct final models.

One strength of Bayesian inference is the necessary transparency that comes from explicitly outlining prior information. In that spirit, our modeling paradigm helps users specify informed prior probability distributions over model sets associated with meaningful extents of spatial covariates—something that is much more difficult to do in competing paradigms. Part of this prior distribution requires identifying the spatial scale for the association of a covariate on a biological response. With some automated approaches, considering models associated with very small increments could be excessive and not scientifically meaningful—and could result in covariates with near 100% correlation, and hence, similar model support between models with covariates from consecutive extents. However, assuming domain knowledge transferred through an informed prior probability will enable posterior results that can shed light on meaningful spatial extents. The Shiny app that we have shared is one way for practitioners to elicit these beliefs and clearly communicate the choices made for which model sets to consider and the use of priors across those sets.

We further acknowledge that ecological processes are often more complicated than the synthetic situations derived in the model selection literature. Spatial processes are often nonlinearly related to ecological outcomes of interest. Spatial processes often interact with each other and require modeling approaches that consider interaction terms and other nonadditive components in modeling frameworks. Automated screening tools, including the one that we have presented here, do not inherently consider these relationships. As with any modeling endeavor, the use of principled workflows can inform modeling choices and note that our framework, in a similar spirit to exploratory data analysis, can serve as the first step in screening spatial covariates.

## Author Contributions

**Andrew Hoegh:** conceptualization (equal), formal analysis (lead), methodology (lead), software (lead), visualization (lead), writing – original draft (lead), writing – review and editing (equal). **Kathryn M. Irvine:** conceptualization (equal), funding acquisition (supporting), writing – review and editing (equal). **Katharine M. Banner:** conceptualization (equal), writing – review and editing (equal). **Luz de Wit:** writing – review and editing (equal). **Brian Reichert:** funding acquisition (lead), writing – review and editing (equal).

## Funding

This work was supported by the CESU agreement (G21AC10748) between USGS‐NOROCK and MSU, G21AC10748.

## Conflicts of Interest

The authors declare no conflicts of interest.

## Supporting information


**Data S1:** ece373271‐sup‐0001‐Supinfo1.pdf.

**Data S2:** ece373271‐sup‐0002‐Supinfo2.xlsx.

**Data S3:** ece373271‐sup‐0003‐Supinfo3.pdf.

**Data S4:** ece373271‐sup‐0004‐Supinfo4.pdf.

**Data S5:** ece373271‐sup‐0005‐Supinfo5.pdf.

**Data S6:** ece373271‐sup‐0006‐Supinfo6.xlsx.

## Data Availability

Data and code used in this manuscript are uploaded as Supporting Information with the submission.

## Endnotes

^1^
Any use of trade, firm, or product names is for descriptive purposes only and does not imply endorsement by the US Government.

## Appendix 1

### Simulation 1 Derivations

Recall from Equation (1) and Equation (2) that $\mathrm{Pr}\left[M_{k}|Y\right]=\frac{\mathrm{Pr}\left[M_{k}\right]\times p\left[Y|M_{k}\right]}{p\left(Y\right)}$, where the marginal likelihood $p\left[Y|M_{k}\right]=\int p\left(Y|\theta ,M_{k}\right)p\left(\theta |M_{k}\right)\mathrm{d\theta}.$ Generally integrating out the parameter vector θ is unfeasible, but in this scenario (in which the variance is known and normal priors on β) it can be solved analytically and, moreover, there are also analytical solutions for the posterior model probabilities. Below we provide details for obtaining $p\left[Y|M_{k}\right]$.

Let

$$p\left[Y|\beta ,\Sigma \right]={\left(2\pi \right)}^{-\frac{n}{2}}{\left|\Sigma \right|}^{-\frac{1}{2}}\exp\left[-\frac{1}{2}{\left(Y-\mathrm{X\beta}\right)}^{\prime }\Sigma^{-1}\left(Y-\mathrm{X\beta}\right)\right]$$

while we avoid explicit notation for covariates associated in model k, all β values could be denoted as $\beta_{\left[k\right]}$, where n is the sample size and

$$p\left[\beta \right]={\left(2\pi \right)}^{-\frac{p}{2}}{\left|T\right|}^{-\frac{1}{2}}\exp\left[-\frac{1}{2}{\left(\beta -\beta_{0}\right)}^{\prime }T^{-1}\left(\beta -\beta_{0}\right)\right]$$

where p is the dimension of the β vector. Note technically, $p\left[\beta \right]=p\left[\beta |M_{K}\right]$, but we follow typical convention and just use $p\left[\beta \right]$. So $\int p\left[Y|\beta ,\Sigma \right]p\left[\beta \right]\mathrm{d\beta}$ can be solved as

=∫2π−n2Σ−12exp−12Y−Xβ′Σ−1Y−Xβ×=∫2π−p2T−12exp−12β−β0′T−1β−β0dβ=2π−n2Σ−12T−12∫2π−p2exp−12Y−Xβ′Σ−1Y−Xβ+β−β0′T−1β−β0dβ=2π−n2Σ−12T−12exp−12Y′Σ−1Y+β0′T−1β0×∫2π−p2exp−12β′X′Σ−1X+T−1β−β′X′Σ−1Y+T−1β0−Y′Σ−1X+β0T−1βdβ

From here, this integration requires completing the square. First we identify that $\mathrm{Var}\left(\beta \right)={\left(X^{\prime }\Sigma^{-1}X+T^{-1}\right)}^{-1}$ and $E\left(\beta \right)={\left(X^{\prime }\Sigma^{-1}X+T^{-1}\right)}^{-1}\left(X^{\prime }\Sigma^{-1}Y+T^{-1}\beta_{0}\right)$. Hence, we are missing the two following terms $\exp\left[-\frac{1}{2}{\left(X^{\prime }\Sigma^{-1}Y+T^{-1}\beta_{0}\right)}^{\prime }{\left(X^{\prime }\Sigma^{-1}X+T^{-1}\right)}^{-1}\left(X^{\prime }\Sigma^{-1}Y+T^{-1}\beta_{0}\right)\right]$ and ${\left|{\left(X^{\prime }\Sigma^{-1}X+T^{-1}\right)}^{-1}\right|}^{-\frac{1}{2}}$. Hence, the integration concludes by adding the missing pieces (and their reciprocals) such that

$$\begin{array}{c} p\left[Y|M_{k}\right]={\left(2\pi \right)}^{-\frac{n}{2}}{\left|\Sigma \right|}^{-\frac{1}{2}}{\left|T\right|}^{-\frac{1}{2}}\exp\left[-\frac{1}{2}\left(Y^{\prime }\Sigma^{-1}Y+\beta_{0}^{\prime }T^{-1}\beta_{0}\right)\right]\times \\ \exp\left[\frac{1}{2}{\left(X^{\prime }\Sigma^{-1}Y+T^{-1}\beta_{0}\right)}^{\prime }{\left(X^{\prime }\Sigma^{-1}X+T^{-1}\right)}^{-1}\left(X^{\prime }\Sigma^{-1}Y+T^{-1}\beta_{0}\right)\right]\times \\ {\left|{\left(X^{\prime }\Sigma^{-1}X+T^{-1}\right)}^{-1}\right|}^{\frac{1}{2}} \end{array}$$
